# Changing weekend effects of air pollutants in Beijing under 2020 COVID-19 lockdown controls

**DOI:** 10.1038/s42949-022-00070-0

**Published:** 2022-09-22

**Authors:** Lingyun Wu, Junfei Xie, Keyu Kang

**Affiliations:** 1 State Key Laboratory of Numerical Modeling for Atmospheric Sciences and Geophysical Fluid Dynamics (LASG), Institute of Atmospheric Physics, Chinese Academy of Sciences, Beijing, 100029 China; 2 Beijing Key Laboratory of Ecological Function Assessment and Regulation Technology of Green Space, Beijing Institute of Landscape Architecture, Beijing, 100102 China; 3 College of Landscape Architecture and Tourism, Hebei Agricultural University, Hebei, 071000 China

**Keywords:** Environmental sciences, Climate sciences, Environmental social sciences

## Abstract

In 2020, lockdown control measures were implemented to prevent a novel coronavirus disease 19 (COVID-19) pandemic in many places of the world, which largely reduced human activities. Here, we detect changes in weekly cycles of PM_2.5_, NO_2_, SO_2_, CO and O_3_ concentrations in 2020 compared to 2018 and 2019 using the observed data at 32 stations in Beijing. Distinct weekly cycles of annual average PM_2.5_, NO_2_, SO_2_ and CO concentrations existed in 2018, while the weekend effects changed in 2020. In addition, the weekly cycle magnitudes of PM_2.5_, NO_2_, SO_2_, and O_3_ concentrations in 2020 decreased by 29.60–69.26% compared to 2018, and 4.49–47.21% compared to 2019. We propose that the changing weekend effects and diminishing weekly cycle magnitudes may be tied to the COVID-19 lockdown controls, which changed human working and lifestyle cycles and reduced anthropogenic emissions of air pollutants on weekends more than weekdays.

## Introduction

The phenomena of weekly cycles were observed early in 1880s^1^, recognized in 1929^2^, and were widely known nowadays^3–14^. The weekly cycles have been attributed to human causes because there is no evidence of 7-day periodicities in nature and stronger weekly cycles are mainly found on urban area with large population density^15–17^. Consequently, the identification of weekly cycles has become a powerful tool for investigating anthropogenic effects on air pollution, meteorological and other variables^18–21^.

A novel coronavirus disease (COVID-19) was firstly reported in late December 2019, and then quickly spread to many places around the world in 2020^22–25^. To prevent the infection of the virus from human mobility, partial or full lockdown control measures were implemented in many countries and regions in 2020^26–29^. The lockdown controls encourage people to stay at home to avoid mass gatherings including reduction of transportation, and closures of school, factories and non-essential businesses and others, which have largely reduced human activities^30–34^.

Many scholars used the unique opportunity to investigate the effects of the lockdown controls on air quality^35–39^. Globally, the concentrations of PM_2.5_, NO_2_, and SO_2_ show reductions, while O_3_ increased with decreased human activities^40–43^. In China, the reduced human activities have been reported to take certain effects on air pollutants^44–49^.

Beijing, the capital of China, homes to 21.89 million population with 87.56% urbanization rate in 2020 (http://nj.tjj.beijing.gov.cn/nj/main/2021tjnj/zk/indexeh.htm). Air pollution has always been an important issue need to be solved with the rapid urbanization in Beijing for recent decades^50–53^, which can produce severe impacts on human health, ecosystem, and economy^54–56^. In early 2020, Beijing suffered COVID-19 pandemic and implemented complete lockdowns from 24 January to 29 April 2020, and partial lockdowns from 30 April to 20 July 2020 to prevent the disease spread. Some studies have reported that lockdown controls in Beijing have largely reduced human activities and related anthropogenic emissions of CO_2_ and air pollutants^57–61^.

Here, the question has been raised if there are distinct changes in weekly cycles of air pollutants under COVID-19 lockdown controls in Beijing. People in Beijing usually work on Monday through Friday and stay at home or take a leisure on Saturday and Sunday. In addition, Beijing enacts traffic restriction based on the last digit on a license plate Monday through Friday. These working cycle and traffic policy lead to obvious weekly cycles of air pollutants. However, COVID-19 lockdown controls have disturbed the working and lifestyle cycles, which would change the weekly cycles of air pollutants.

In this study, we aim to detect changes in weekly cycles of air pollutants in Beijing under 2020 COVID-19 lockdown controls compared to 2018 and 2019. We investigate not only changes in weekly cycles of primary pollutants of PM_2.5_, NO_2_, SO_2_, CO but also secondary pollutants of O_3_. Our analysis is not restricted lockdown control periods but is basis of the yearly average values because the COVID-19 lockdown controls not only affect control periods but also the following period. In addition, the yearly average can reduce the effects of seasonal change. Observational data at 32 stations were used to compare the weekly cycle changes, which cover all districts of Beijing with different land surface and pollution emissions (Supplementary Fig. 1 and Table 1). Moreover, we apply student’s *t*-test and Wilcoxon rank-sum test to evaluate the significance of weekend effects. Further analyses attempt to reveal the relation of weekend effect changes with meteorological conditions and human activities. The main goal of the study is to present evidence that the weekly cycles are a product of human activities in a convincing way and provide some thoughts of how to make a plan for improving air quality on urban area in future.

**Table 1:** Descriptions of 32 stations in Beijing.

	Station Name	Location	Longitude	Latitude	Elevation (m)
A1	Dongsi (DS)	Dongcheng District Environmental Protection Bureau (Block in urban)	116.417	39.929	31
A2	Tiantan (TT)	Inside Temple of Heaven Park (Park in urban)	116.407	39.886	33
A3	Guanyuan (GY)	Beijing Municipal Party School (School in urban)	116.339	39.929	47
A4	Wanshouxigong (WSXG)	Inside Wanshou Park (Park in urban)	116.352	39.878	51
A5	Aotizhongxin (ATZX)	Inside Olympic Center (Sports Center in urban)	116.397	39.982	42
A6	Nongzhanguan (NZG)	Residential Area of Agricultural Exhibition Hall (Residential zone in urban)	116.461	39.937	43
A7	Beibuxinqu (BBXQ)	Inside Daoxiang Lake Park (Park in urban)	116.174	40.09	37
A8	Wanliu (WL)	Inside Changchun Fitness Park (Park in urban)	116.287	39.987	44
A9	Fengtaihuayan (FTHY)	Inside Fengtai Garden (Park in urban)	116.279	39.863	54
A10	Yungang (YG)	Urban Area of Fengtai District (Block in urban)	116.146	39.824	106
A11	Gucheng (GC)	Shijingshan Environmental Protection Bureau (Institution in urban)	116.184	39.914	71
B1	Fangshan (FS)	Urban Area of Fangshan (Block in town)	116.136	39.742	44
B2	Daxing (DX)	Urban Area of Daxingcheng (Block in town)	116.404	39.718	27
B3	Yizhuang (YZ)	Urban Area of Yizhuang (Block in town)	116.506	39.795	17
B5	Shunyi (SY)	Urban Area of Shunyi (Block in town)	116.655	40.127	15
B6	Changping (CP)	Urban Area of Changping (Block in town)	116.23	40.217	74
B7	Mentougou (MTG)	Inside Binhe Century Plaza Park (Park in town)	116.106	39.937	113
B8	Yanqing (YQ)	Urban Area of Yanqing (Block in town)	115.972	40.453	503
B9	Pinggu (PG)	Urban Area of Pinggu (Block in town)	117.1	40.143	17
B10	Miyun (MY)	Urban Area of Miyun (Block in town)	116.832	40.37	73
B11	Huairou (HR)	Urban Area of Huairou (Block in town)	116.628	40.328	52
C1	Dingling (DL)	Dingling, Changping (Park in suburban)	116.22	40.292	231
D1	Yufa (YF)	Southern Beijing (suburban)	116.3	39.52	15
D2	Miyunshuiku (MYSK)	Northeast Beijing (suburban)	116.911	40.499	151
D3	Badaling (BDL)	Northwestern Beijing (suburban)	115.988	40.365	530
D4	Yongledian (YLD)	Southeastern Beijing (suburban)	116.783	39.712	12
D5	Liulihe (LLH)	Southwestern Beijing (suburban)	116	39.58	26
D6	Donggaocun (DGC)	Eastern Beijing (suburban)	117.12	40.1	57
E1	Yongdingmennei (YDMN)	Yongdingmen Inner Street (Side of the street)	116.394	39.876	33
E2	Nansanhuan (NSH)	Next to South Third Ring Western Road (Side of the street)	116.368	39.856	49
E3	Dongsihuan (DSH)	Next to East Fourth Ring North Road (Side of the street)	116.483	39.939	34
E4	Xizhimenbei (XZMB)	Xizhimen North Street (Side of the street)	116.349	39.954	45

A1–A11: Urban environmental assessment points.

B1–B11: Suburban environmental assessment points.

C1: Background control points.

D1–D5: Regional transmission monitor points.

E1–E4: Traffic pollution control points.

## Results

### Changes in the annual average air pollutants

**Fig. 1:**
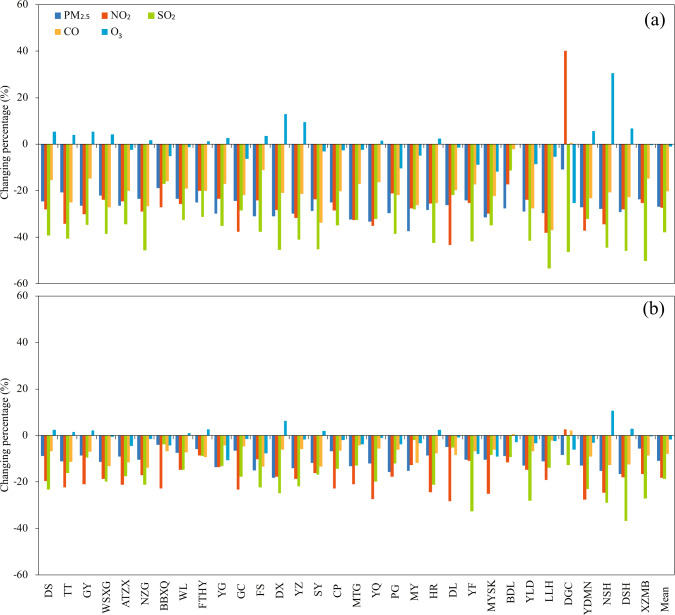
The changes in the annual average air pollutants. The changing percentages of annual average PM_2.5_, NO_2_, SO_2_, CO and O_3_ concentrations in 2020 compared to (**a**) 2018 and (**b**) 2019.

Figure 1 shows changing percentages of annual average air pollutants in 2020 compared to the previous 2 years. The annual average PM_2.5_ and SO_2_ concentrations at 32 stations in Beijing in 2020 were all lower than those in 2018 and 2019. The annual average NO_2_ and CO concentrations in 2020 were also lower than those in 2018 and 2019 except for station of Donggaocun. For O_3_ concentration, there were about half stations showing higher values in 2020 than 2018 and 2019. On average of 32 stations, PM_2.5_, NO_2_, SO_2_, CO, and O_3_ concentrations were 38.13 μg m^−3^, 29.82 μg m^−3^, 3.70 μg m^−3^, 0.64 mg m^−3^, and 59.80 μg m^−3^ in 2020 and decreased by 26.75%, 27.31%, 37.87%, 20.20%, and 0.99% compared to 2018 and decreased by 10.86%, 18.20%, 18.76%, 7.94%, and 1.78% to 2019 (Table 2).

**Table 2:** The annual average PM_2.5_, NO_2_, SO_2_, CO and O_3_ concentrations of 32 stations in Beijing in 2018, 2019, and 2020.

Year	PM_2.5_ (μg m^−3^)	NO_2_ (μg m^−3^)	SO_2_ (μg m^−3^)	CO (mg m^−3^)	O_3_ (μg m^−3^)
2018	52.06	41.02	5.95	0.81	60.39
2019	42.78	36.45	4.55	0.70	60.88
2020	38.13	29.82	3.70	0.64	59.80

### Changes in weekly cycle of air pollutants

**Fig. 2:**
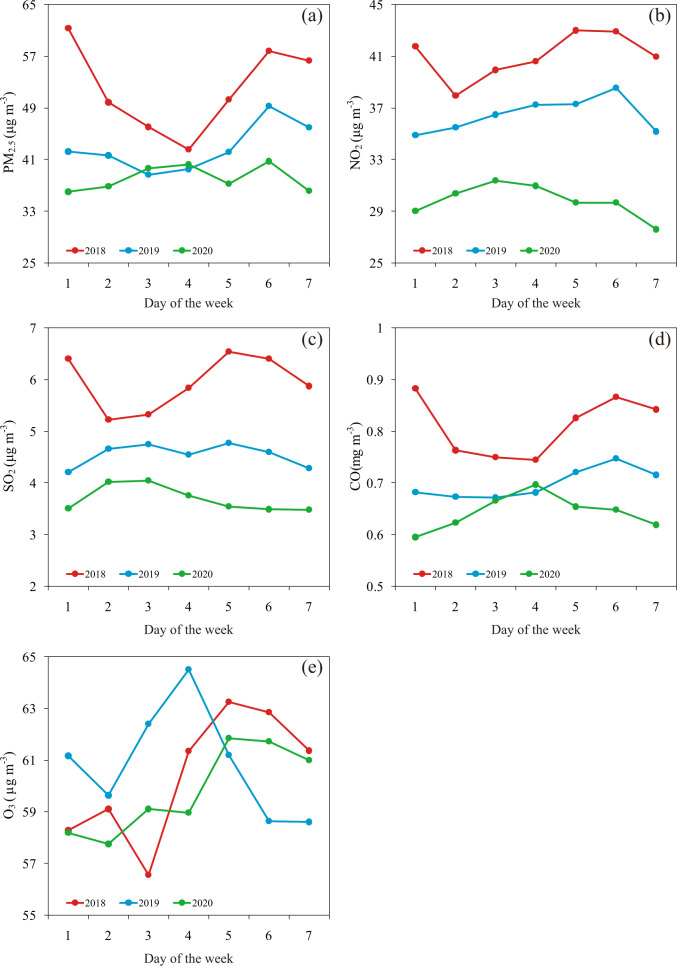
Air pollutants by day of the week. The annual average (**a**) PM_2.5_, (**b**) NO_2_, (**c**) SO_2_, (**d**) CO and (**e**) O_3_ concentrations by day of the week for the mean of 32 stations in Beijing in 2018, 2019, and 2020.

Figure 2 presents the annual average air pollutants of 32 stations for each day of the week in 2018, 2019, and 2020. In 2018 and 2019, PM_2.5_ time series at most of stations consistently exhibited an evident 7-day cycle: a downward trend from Monday to Thursday/Wednesday, and then rising to Saturday (Fig. 2a and Supplementary Fig. 2). In contrast, no indications of these distinct weekly cycles could be found in 2020. Instead, Saturday was the day of highest PM_2.5,_ followed rather closely by Thursday and Wednesday, while Monday and Sunday were the days of lowest values in 2020. Both NO_2_ and SO_2_ concentrations in 2018 exhibited higher values on Friday through Monday, while the larger values occurred from Tuesday through Thursday in 2020 (Fig. 2b, c and Supplementary Figs. 3–4). The seven-day cycle of CO concentrations show very similar patterns to those of PM_2.5_ in 2018 and 2019, while the weekly cycle in 2020 show almost opposite pattern (Fig. 2d and Supplementary Fig. 5). By contrast, O_3_ concentrations have different weekly cycle patterns from other air pollutants: they have relatively higher values on Friday through Sunday in 2020, which were similar to those in 2018 but quite different to those in 2019 (Fig. 2e and Supplementary Fig. 6).

### Changes in weekend effects of air pollutants

In previous studies, weekend and weekday have different definitions depending on the signal of weekly cycle. For instance, Simmonds and Keay^62^ defined Saturday through Sunday as the weekend and Monday through Friday as the weekday. While, some studies^63–65^ took Saturday through Monday as the weekend and Wednesday through Friday as the weekday. In the present study, the larger values of PM_2.5_, NO_2_, SO_2_, and CO in 2018 and 2019 mainly appeared on Friday through Monday. Thereby, we define Friday through Monday as the weekend and Tuesday through Thursday as the weekday to evaluate the changes of weekend effects in 2020 relative to 2018 and 2019. Here, we follow Daniel et al.’s method^65^ to evaluate the weekend effects and weekly cycle intensities by using weekend effect magnitude (the difference between weekend and weekdays WEM, see Eq. 1 in METHODS) and weekly cycle magnitude (weekly maximum minus weekly minimum, WCM, see Eq. 2 in METHODS).

**Fig. 3:**
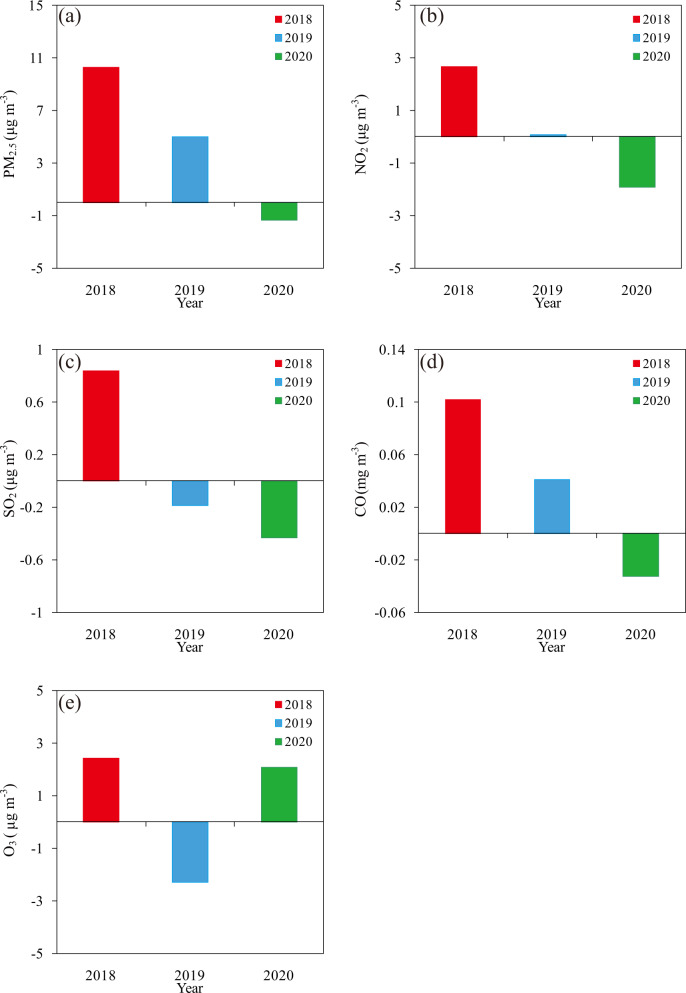
Weekend effect magnitudes of air pollutants. The annual average (**a**) PM_2.5_, (**b**) NO_2_, (**c**) SO_2_, (**d**) CO and (**e**) O_3_ concentrations weekend effect magnitudes for the mean of 32 stations in Beijing in 2018, 2019, and 2020. The weekend refers to Friday through Monday, and weekday is Tuesday through Thursday. The weekend effect magnitude is computed by subtracting the average weekday value from the average weekend value.

Supplementary Figs. 7–11 show the WEM of PM_2.5_, NO_2_, SO_2_, CO and O_3_ concentrations for each year at 32 stations in Beijing. The weekend values of PM_2.5_ were greater than weekdays at all stations in 2018 and 2019 with the values ranging from 7.05 to 14.38 μg m^−3^ and 1.98 to 8.72 μg m^−3^. In 2020, only 3 stations showed larger values on weekend than weekdays, and the WEM fluctuated between −4.10 and 1.14 μgm^−^^3^ (Supplementary Fig. 7). The weekend-weekday differences were significant at the 90% confidence level by student’s *t*-test and Wilcoxon rank-sum test at 21 stations in 2018 and 16 stations in 2019, respectively (Table S1). By contrast, there are no any stations showing significant weekend-weekday differences at the 90% confidence level by student’s *t*-test and Wilcoxon rank-sum test in 2020. On the average of 32 stations, the WEM of PM_2.5_ in 2018 and 2019 were 10.30 μg m^−3^ and 4.98 μg m^−3^ higher on weekend than weekdays and the differences between weekends and weekdays are both at the 90% significant confidence level by student’s *t*-test and Wilcoxon rank-sum test (Fig. 3a). Comparatively, weekend was smaller 1.38 μg m^−3^ than weekdays and the difference was insignificant by student’s *t*-test and Wilcoxon rank-sum test in 2020. The results show that weekends and weekdays do not differ from each other in Beijing in 2020, implying that the weekend effects of the annual average PM_2.5_ vanished under lockdown control measures.

The values of NO_2_ and SO_2_ concentrations on weekend were larger than weekdays at 32 and 31 stations and the weekend-weekday difference were significant at the 90% confidence level by Student’s *t*-test and Wilcoxon rank-sum test at 14 stations in 2018 (Supplementary Figs. 8–9, Tables S2–3). In 2020, NO_2_ and SO_2_ concentrations on weekend were smaller than weekdays at 32 and 28 stations and the differences were significant at the 90% significant confidence level by Student’s *t*-test and Wilcoxon rank-sum test at 19 and 25 stations. On average of 32 stations, NO_2_ and SO_2_ concentrations on weekend were larger 2.66 μg m^−3^ and 0.84 μg m^−3^ than weekday in 2018 at the 90% significant confidence level by Student’s *t*-test and Wilcoxon rank-sum test (Fig. 3b, c). In 2020, NO_2_ and SO_2_ concentrations on weekends were lower 1.91 μg m^−3^ and 0.43 μg m^−3^ than weekdays and the differences were at the 90% significant confidence level by student’s *t*-test and Wilcoxon rank-sum test.

The weekend values of CO were greater than weekdays at all stations in 2018 and 2019 while there were only 2 stations showing larger values on weekends than weekdays in 2020 (Supplementary Fig. 10). Student’s *t*-test and Wilcoxon rank-sum test reveal that the weekend-weekday differences were significant at the 90% confidence level at 28 stations in 2018 and 18 stations in 2019, respectively (Table S4). By contrast, there are only 1 station showing the 90% significance of difference by student’s t-test and Wilcoxon rank-sum test in 2020. On the average of 32 stations, CO were 0.10 mg m^−3^ and 0.04 mg m^−3^ higher on weekend than weekdays and the differences between weekends and weekdays are both at the 90% significant confidence level by student’s *t*-test and Wilcoxon rank-sum test in 2018 and 2019 (Fig. 3d). Comparatively, weekend was smaller 0.03 mg m^−3^ than weekdays and the difference was insignificant by student’s *t*-test and Wilcoxon rank-sum test in 2020. Similar to PM_2.5_, the weekend effects of the annual average CO in 2018 and 2019 vanished in 2020.

The weekend values of O_3_ were greater than weekdays at 30 stations in 2018 and 32 stations in 2020 (Supplementary Fig. 11). The weekend-weekday differences were significant at the 90% confidence level by student’s *t*-test and Wilcoxon rank-sum test at 5 and 8 stations in 2018 and 2020 (Table S5). On the average of 32 stations, the WEM in 2018 and 2020 were 2.43 μg m^−3^ and 2.09 μg m^−3^ higher on weekend than weekdays and the differences were both insignificant by student’s *t*-test and Wilcoxon rank-sum test (Fig. 3e).

Conclusively, the annual average PM_2.5_, NO_2_, SO_2_, and CO concentrations on weekends were higher than weekdays in 2018, but they showed opposite pattern in 2020. Further analysis reveals that the reductions of PM_2.5_, NO_2_, SO_2_, and CO on the weekends were larger than weekdays in 2020 relative to 2018, and 2019 (Table 3). In other words, the changing weekend effects of PM_2.5_, NO_2_, SO_2_, and CO in 2020 are mainly attributed to more reductions on the weekends than weekdays.

**Table 3:** The averaged reduction percentages of PM_2.5_, NO_2_, SO_2_, CO, and O_3_ concentrations for 32 stations on weekends and weekdays in Beijing in 2020 relative to 2018 and 2019.

	Relative to 2018		Relative to 2019	
	weekends	weekdays	weekends	weekdays
PM_2.5_	33.51	15.69	16.44	2.56
NO_2_	31.24	23.61	20.53	15.13
SO_2_	44.37	27.90	21.49	15.31
CO	26.36	12.04	12.20	2.01
O_3_	1.22	0.68	1.32	5.75

### Diminishing weekly cycle magnitude of air pollutants

**Fig. 4:**
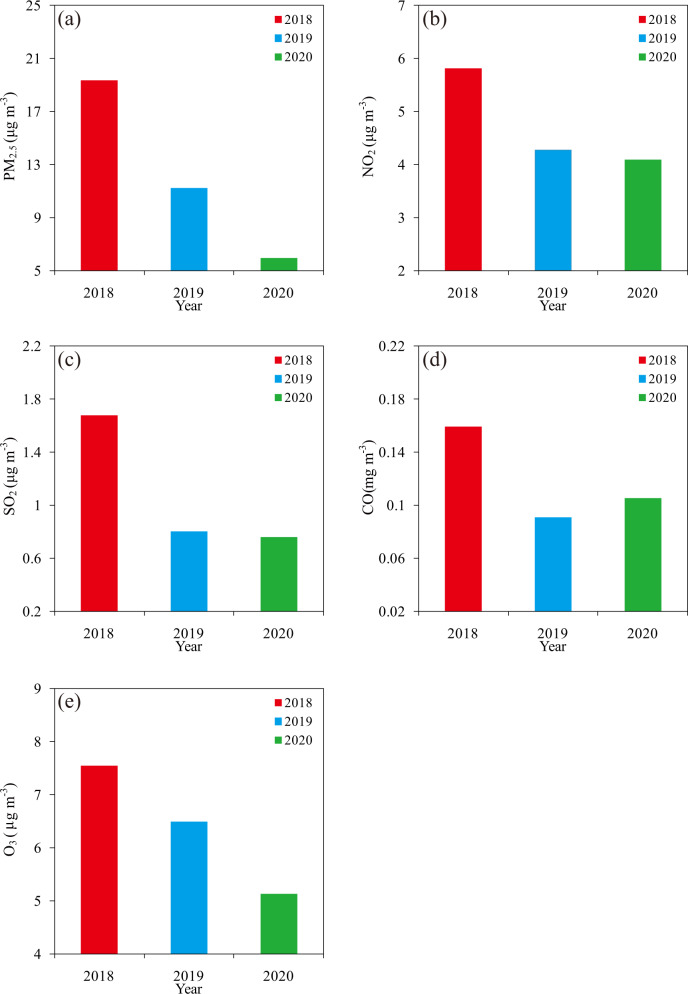
Weekly cycle magnitudes of air pollutants. The annual average (**a**) PM_2.5_, (**b**) NO_2_, (**c**) SO_2_, (**d**) CO and (**e**) O_3_ concentrations weekly cycle magnitudes for the mean of 32 stations in Beijing in 2018, 2019, and 2020. The weekly cycle magnitude is defined as the difference between the weekly maximum and minimum.

Moreover, we detect the changes in WCMs of PM_2.5_, NO_2_, SO_2_, CO and O_3_ concentrations in 2020 compared to the previous 2 years (Fig. 4 and Supplementary Figs. 12–16). The WCMs of PM_2.5_ concentrations in 2020 were smaller than those in 2018 and 2019 at nearly all stations (Supplementary Fig. 12). The average WCMs of PM_2.5_ concentrations for 32 stations in 2018 and 2019 were 19.31 and 11.20 μg m^−3^, while the average value in 2020 was only 5.94 μg m^−3^ and decreased by 69.24% and 47.21%. The average WCM of NO_2_, SO_2_, and O_3_ for 32 stations in 2020 decreased by 29.69%, 54.82%, and 32.05% compared to 2018, and 4.49%, 5.27%, and 21.05% to 2019. These results demonstrate that WCMs of air pollutants largely diminished in 2020 compared to the previous 2 years. We notice that the WCM of CO concentrations in 2020 decreased 33.97% compared to 2018, but increased by 15.96% compared to 2019.

## Discussions

Lockdown controls in Beijing during the COVID-19 pandemic in 2020 provide a real experiment to investigate the effects of human activities on weekly cycles of air pollutants. The annual average PM_2.5_, NO_2_, SO_2_, CO, and O_3_ concentrations of 32 stations in 2020 in Beijing were lower than those in 2018 and 2019, and decreased by 0.99–37.87% compared to 2018 and 1.78–18.76% to 2019. A clear PM_2.5_, NO_2_, SO_2_ and CO weekly cycle existed with a noticeable weekend higher values than weekdays in 2018. However, the patterns of weekend effects were almost opposite in 2020. Meanwhile, the weekend cycle magnitudes of PM_2.5_, NO_2_, SO_2_, and O_3_ concentrations in 2020 largely decreased compared to those in 2018 and 2019.

**Fig. 5:**
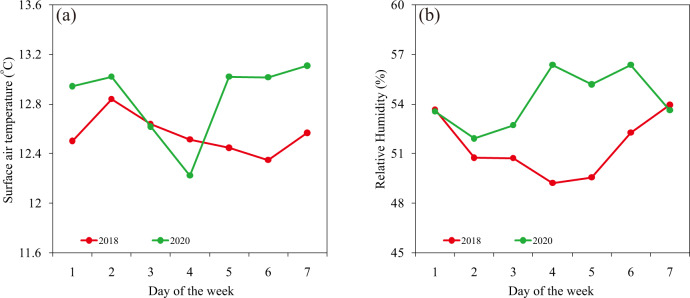
Meteorological conditions by day of the week. The annual average (**a**) surface air temperature and (**b**) relative humidity by day of the week of for the mean of 14 stations in Beijing in 2018 and 2020.

The changing air pollutants weekend effects in Beijing in 2020 is a key finding in this study. One possible factor is meteorological conditions^66,67^. The surface air temperature shows slightly lower value on weekends than weekdays in 2018, while they have reverse patterns in 2020 (Fig. 5a). The higher annual average relative humidity occurred on Sunday and Monday in 2018, and they appeared from Thursday through Saturday in 2020 (Fig. 5b). The weekly cycles of surface air temperature and relative humidity were quite different from those of air pollutants (Fig. 2). The differences between weekends and weekdays were significant at the 90% confidence level by the Student’s *t*-test but they were insignificant by Wilcoxon rank-sum test. The values of relative humidity were both higher on weekends than weekdays in 2018 and 2020 but the difference were insignificant by student’s *t*-test and Wilcoxon rank-sum test. These suggest that meteorological conditions cannot explain changing weekend effects of air pollutants in a satisfying way.

Another possible factor is COVID-19 lockdown control. The anthropogenic sources of air pollutants include emissions from transportation, industrial activity, residential combustions and agriculture^68,69^. Lockdown controls enact closures of schools, libraries, museums, factories, commercial events and others, thus change the air pollutant emissions of weekly working and lifestyle cycles from transportation, industrial activities and others. For instance, passenger traffic and passenger turnover in Beijing in 2020, decreased by 49.7% and 55.8%, and public transport by bus and subway rail decreased by 41.7% and 42.1% compared to 2019 (Table 4). Here, we compared the weekly cycles of CO_2_ emissions from ground transport in China in 2020 to those in 2019. We found that CO_2_ emissions from ground transport decreased by 12.69%, and reductions on weekends and weekdays were 14.73% and 10.04%, which suggesting that more reduced human activities from weekends.

**Table 4:** Decrease of passenger transport in 2020 over 2019.

Item	Decrease over the previous year (%)
Total passenger traffic	49.7
Passenger turnover	55.8
Passenger capacity in public transportation	41.7
Passenger capacity in subway lines	42.1

We detect diminishing weekly cycle magnitudes and changing weekend effects of air pollutants simultaneously with large decreasing in transportation due to lockdown controls under COVID-19 in Beijing in 2020. Therefore, we propose that the lockdown controls, which reduced the anthropogenic emissions and change the working and lifestyle cycles, are the probable reason for changing weekend effects and diminishing weekly cycle magnitudes in Beijing. Ashworth^2^ found that the precipitation weekend effect vanished when factories were in operation for seven days of week during the World War I years. In very recent, vanishing weekly hydropeaking cycles were observed in American and Canadian rivers^70^. Combined these studies with our results together, we can conclude that a weekly cycle is an obvious signature of anthropogenic working cycles and the changing air pollutants weekend effects may mainly attribute to lockdown controls in 2020.

## Methods

### Data sources

The observed hourly PM_2.5_, PM10, SO_2_, NO_2_, CO, and O_3_ concentration data at 34 stations in 2018, 2019, and 2020 were achieved from the Beijing Environmental Protection Monitoring Center (http://www.bjmemc.com.cn/). The 34 stations cover all urban, suburban, and rural districts of Beijing with different land surface and air pollutant source emissions. In general, the 34 stations are classified into 5 types based on their monitoring functions including urban environmental assessment points, suburban environmental assessment points, background control points, regional transmission monitor points, and traffic pollution control points. The elevations of station are below 100 m at 28 stations, between 100 and 200 m at 4 stations and above 500 m at 2 stations. There are a lot of missing data of PM10 at all stations in 2019 and 2020, and many missing data of O_3_, SO_2_, and NO_2_ at Tongzhou and Qianmen stations in 2020. For the reliability of the results, PM_2.5_, SO_2_, NO_2_, CO, and O_3_ at 32 stations in 2018, 2019, and 2020 were analyzed in this study (Supplementary Fig. 1 and Table 1).

The data of total passenger traffic, passenger turnover, passenger capacity in public transportation, and passenger capacity in subway lines were obtained from Statistical communique on the national economy and social development of Beijing in 2019 and 2020 (http://tjj.beijing.gov.cn/EnglishSite/SC/[2021-10-16]) and Beijing Statistical Yearbooks in 2021 (http://nj.tjj.beijing.gov.cn/nj/main/2021-tjnj/zk/indexeh.htm).

Daily CO_2_ in 2019 and 2020 emission data in China were obtained from http://www.carbonmonitor.org or http://www.carbonmonitor.org.cn. The calculation of CO_2_ emissions cover four sectors of power generation, industry, transportation and household consumption. The CO_2_ emissions from ground transportation were estimated by a sigmoid function with TomTom congestion data^32^.

Hourly mean surface air temperature and relative humidity data in 2018 and 2020 were from Beijing Meteorological Service. The data were collected from 14 observational stations including Changping, Chaoyang, Daxing, Fangshan, Fengtai, Haidian, Huairou, Mentougou, Miyun, Pinggu, Shijingshan, Shunyi, Tongzhou, and Yanqing stations.

### Weekend effect magnitude and weekly cycle magnitude

In this study, we define Friday through Monday as the weekend, and Tuesday through Thursday as weekday. The weekend effect magnitude (WEM) is computed by subtracting the average weekday value (*w*_*d*_) from the average weekend value (*w*_*e*_) (Eq. 1). The weekly cycle magnitude (WCM) is defined as the difference between the weekly maximum (*w*_*max*_) and minimum (*w*_*min*_) (Eq. 2).

1 $$WEM = w_d - w_e$$

2 $$WCM = w_{{{{\mathrm{max}}}}} - w_{\min }$$

### Statistical analysis

In this study, we performed student’s t-test and Wilcoxon rank-sum test to detect the significance of weekend effects of air pollutants and meteorological conditions (see RESULTS and DICUSSIONS Sections). Student’s *t*-test is commonly used to evaluate the significance of weekend effects and weekly cycle magnitudes^8,9,64–66^. The test can check whether two groups have different means. Wilcoxon rank-sum test is another effective method to reveal if weekend effect is significant. Wilcoxon rank-sum test is a non-parametric approach without limiting to a normal distribution, therefore, it is more flexible than Student’s *t*-test^66^.

## Supplementary information

Supplementary Information

## Data Availability

The observed hourly PM_2.5_, PM10, SO_2_, NO_2_, CO, and O_3_ concentration data at 32 stations in 2018, 2019, and 2020 were achieved from the Beijing Environmental Protection Monitoring Center (http://www.bjmemc.com.cn/). The data of total passenger traffic, passenger turnover, passenger capacity in public transportation, and passenger capacity in subway lines were obtained from Statistical communique on the national economy and social development of Beijing in 2019 and 2020 (http://tjj.beijing.gov.cn/EnglishSite/SC/[2021-10-16]) and Beijing Statistical Yearbooks in 2021 (http://nj.tjj.beijing.gov.cn/nj/main/2021-tjnj/zk/indexeh.htm). Daily CO_2_ in 2019 and 2020 emission data in China were obtained from http://www.carbonmonitor.org or http://www.carbonmonitor.org.cn. Hourly mean surface air temperature and relative humidity data in 2018 and 2020 were from Beijing Meteorological Service.
