# Supplementary material for: Changing weekend effects of air pollutants in Beijing under 2020 COVID-19 lockdown controls
Source: NPJ Urban Sustain. 2022 Sep 22;2(1):23. doi: 10.1038/s42949-022-00070-0 (PMC9510312; doi:10.1038/s42949-022-00070-0)
Supplement: Supplementary file 1 — Supplementary Information [file 42949_2022_70_MOESM1_ESM.pdf]

Supplementary Information

Changing weekend effects of air pollutants in Beijing under 2020 COVID-19 lockdown controls

Lingyun Wu[1], Junfei Xie[2]* and Keyu Kang[3]

1 State Key Laboratory of Numerical Modeling for Atmospheric Sciences and Geophysical Fluid Dynamics (LASG), Institute of Atmospheric Physics, Chinese Academy of Sciences, Beijing 100029, China

2 Beijing Key Laboratory of Ecological Function Assessment and Regulation Technology of Green Space, Beijing Institute of Landscape Architecture, Beijing 100102, China

3 College of Landscape Architecture and Tourism, Hebei Agricultural University, Hebei 071000, China

Supplementary Table 1

Supplementary Table 2

Supplementary Table 3

Supplementary Table 4

Supplementary Table 5

Supplementary Figure 1

Supplementary Figure 2

Supplementary Figure 3

Supplementary Figure 4

Supplementary Figure 5

Supplementary Figure 6

Supplementary Figure 7

Supplementary Figure 8

Supplementary Figure 9

Supplementary Figure 10

Supplementary Figure 11

Supplementary Figure 12

Supplementary Figure 13

Supplementary Figure 14

Supplementary Figure 15

Supplementary Figure 16

Supplementary Table 1 P-value for difference between weekends and weekdays of $PM_{2.5}$ at 32 stations in 2018, 2019, and 2020 by student's *t*-test

Stations	2018	2019	2020
DS	**0.0260***	0.0919	0.2689
TT	**0.0269***	**0.0654***	0.4979
GY	0.0368	**0.0752***	0.5795
WSXG	**0.0194***	**0.0520***	0.5420
ATZX	0.0348	0.1083	0.5175
NZG	**0.0290***	**0.0778***	0.2740
BBXQ	**0.0234***	0.3730	0.8391
WL	0.0326	0.1760	0.8809
FTHY	**0.0106***	**0.0388***	0.9001
YG	**0.0110***	**0.0590***	0.4003
GC	**0.0150***	**0.0461***	0.5931
FS	**0.0077***	**0.0081***	0.6004
DX	**0.0072***	**0.0216***	0.1042
YZ	**0.0044***	**0.0214***	0.0309
SY	0.0718	0.1659	0.9230
CP	0.0870	0.2117	0.2175
MTG	**0.0259***	0.2147	0.4148
YQ	**0.0348***	0.4139	0.0841
PG	0.1386	0.0456	0.2109
MY	0.1931	0.2736	0.5383
HR	0.1305	0.2350	0.9699
DL	0.0971	0.1257	0.1490
YF	**0.0030***	**0.0303***	0.4148
MYSK	0.1973	0.2310	0.9459
BDL	**0.0482***	0.4198	0.4466
YLD	**0.0070***	**0.0385***	0.3247
LLH	**0.0041***	**0.0048***	0.7296
DGC	0.0752	**0.0429***	0.7926
YDMN	**0.0123***	**0.0667***	0.4026
NSH	**0.0069***	0.0674	0.1564
DSH	**0.0289***	**0.0807***	0.0491
XZMB	**0.0232***	0.1004	0.4583
Mean	**0.0246***	**0.0689***	0.4195

* denotes that weekend and weekday difference is significant at the 90% confidence level by Wilcoxon rank-sum test

Number in bold denotes that weekend and weekday difference is significant at the 90% confidence level by student's *t*-test and Wilcoxon rank-sum test

Supplementary Table 2. P-value for difference between weekends and weekdays of NO_2 at 32 stations in 2018, 2019, and 2020 by student's *t*-test

Stations	2018	2019	2020
DS	0.3236	0.797	**0.0121***
TT	0.2775	0.9716	**0.0398***
GY	0.1157	0.9412	**0.0333***
WSXG	0.0842	0.8692	0.0993
ATZX	**0.0477***	0.6224	**0.0040***
NZG	0.1779	0.9486	**0.0068***
BBXQ	**0.0333***	0.9045	0.1201
WL	0.0594	0.8898	**0.0998***
FTHY	0.0771	0.8626	0.0981
YG	**0.0038***	0.7333	**0.0408***
GC	**0.0013***	0.8938	0.1742
FS	**0.0085***	0.8680	**0.0655***
DX	0.0413	0.9337	**0.0322***
YZ	0.3076	0.6080	**0.0081***
SY	0.0816	0.8402	0.0795
CP	**0.0072***	0.6279	**0.0215***
MTG	**0.0003***	0.8433	0.2850
YQ	0.1080	0.6980	0.7589
PG	0.0343	0.2251	0.0459
MY	0.0744	0.4815	0.4680
HR	**0.0870***	0.3997	0.1459
DL	**0.0008***	0.3215	**0.0284***
YF	**0.0284***	0.4657	**0.0278***
MYSK	**0.0006***	0.2043	0.1376
BDL	**0.0680***	0.6699	**0.0181***
YLD	0.0951	0.3635	**0.0122***
LLH	**0.0256***	**0.0306***	**0.0214***
DGC	**0.0029***	0.9798	0.1258
YDMN	0.5139	0.7843	**0.0686***
NSH	0.1881	0.3125	0.1095
DSH	0.3694	0.8291	**0.0601***
XZMB	0.3557	0.1461	**0.0993***
Mean	**0.0295***	0.9526	**0.0287***

* denotes that weekend and weekday difference is significant at the 90% confidence level by Wilcoxon rank-sum test

Number in bold denotes that weekend and weekday difference is significant at the 90% confidence level by student's *t*-test and Wilcoxon rank-sum test

Supplementary Table 3. P-value for difference between weekends and weekdays of SO_2 at 32 stations in 2018, 2019, and 2020student's *t*-test

Stations	2018	2019	2020
DS	0.0354	0.6683	**0.0121***
TT	0.0189	0.2056	**0.0349***
GY	0.0384	0.4961	**0.0148***
WSXG	**0.0078***	0.4494	**0.0118***
ATZX	**0.0127***	0.9119	**0.0084***
NZG	**0.0219***	0.5402	**0.0307***
BBXQ	0.0466	0.2469	**0.0549***
WL	0.0856	0.984	0.0814
FTHY	**0.0136***	0.8879	**0.0165***
YG	**0.0296***	0.2651	**0.0016***
GC	0.1142	0.531	0.111
FS	**0.0265***	0.5554	**0.0057***
DX	**0.0018***	0.8569	**0.0193***
YZ	**0.0101***	0.469	**0.0063***
SY	**0.0236***	0.2261	**0.0087***
CP	0.0485	0.1447	**0.0380***
MTG	0.0358	0.3595	0.1086
YQ	0.1889	0.0667	0.1025
PG	0.1998	0.5141	**0.0486***
MY	**0.0077***	0.6371	0.0626
HR	**0.0490***	0.1086	**0.0122***
DL	**0.0170***	0.4666	**0.0176***
YF	**0.0068***	0.2741	**0.0001***
MYSK	0.4316	0.3963	0.0487
BDL	0.3967	0.7317	**0.0564***
YLD	0.0258	0.2182	**0.0239***
LLH	0.1906	0.3977	0.2963
DGC	0.0516	0.6541	**0.0131***
YDMN	0.0606	0.2517	**0.0027***
NSH	0.0375	0.4464	**0.0139***
DSH	0.2147	0.4312	**0.0032***
XZMB	**0.0056***	0.2771	**0.0250***
Mean	**0.0166***	0.3076	**0.0027***

* denotes that weekend and weekday difference is significant at the 90% confidence level by Wilcoxon rank-sum test

Number in bold denotes that weekend and weekday difference is significant at the 90% confidence level by student's t-test and Wilcoxon rank-sum test

Supplementary Table 4. P-value for difference between weekends and weekdays of CO at 32 stations in 2018, 2019, and 2020student's *t*-test

Stations	2018	2019	2020
DS	**0.0014***	**0.0533***	0.1285
TT	**0.0019***	**0.0365***	0.0618
GY	**0.0000***	**0.0444***	0.1388
WSXG	**0.0011***	**0.0363***	0.1779
ATZX	**0.0003***	0.1361	0.1118
NZG	**0.0010***	0.1583	0.1385
BBXQ	**0.0009***	0.4261	0.227
WL	**0.0024***	0.1242	0.4622
FTHY	**0.0006***	**0.0478***	0.1552
YG	**0.0017***	**0.0094***	0.3393
GC	**0.0002***	**0.0432***	0.3779
FS	**0.0069***	**0.0007***	0.6158
DX	**0.0107***	**0.0038***	0.2074
YZ	**0.0016***	**0.0221***	0.0684
SY	**0.0162***	0.1794	0.5162
CP	**0.0085***	0.3662	0.2065
MTG	**0.0006***	0.5316	0.3972
YQ	**0.0007***	0.2115	0.3764
PG	0.1189	**0.0191***	0.0634
MY	0.0563	0.4759	0.8114
HR	**0.0329***	0.2491	0.9106
DL	**0.0027***	0.5132	0.1858
YF	**0.0203***	**0.0349***	0.5137
MYSK	0.0575	0.1895	0.9801
BDL	**0.0006***	0.3996	0.353
YLD	**0.0026***	0.0794	**0.0126***
LLH	**0.0211***	**0.0230***	0.9955
DGC	0.0431	**0.0274***	0.1964
YDMN	**0.0038***	**0.0853***	0.1275
NSH	**0.0022***	**0.0119***	0.3138
DSH	**0.0044***	**0.0446***	0.0976
XZMB	**0.0002***	**0.0072***	0.0552
Mean	**0.0013***	**0.0503***	0.2335

* denotes that weekend and weekday difference is significant at the 90% confidence level by Wilcoxon rank-sum test

Number in bold denotes that weekend and weekday difference is significant at the 90% confidence level by student's t-test and Wilcoxon rank-sum test

Supplementary Table 5. P-value for difference between weekends and weekdays of O_3 at 32 stations in 2018, 2019, and 2020student's *t*-test

Stations	2018	2019	2020
DS	0.2067	0.2331	**0.0102***
TT	0.2659	0.3676	**0.0185***
GY	0.4056	0.1529	0.0561
WSXG	0.3763	0.3399	0.0375
ATZX	0.1410	0.0829	0.0671
NZG	0.2499	0.3034	**0.0273***
BBXQ	0.8431	0.1222	0.2409
WL	0.3128	0.0779	0.1779
FTHY	0.3768	0.1296	0.0742
YG	0.6914	0.2023	**0.0246***
GC	0.6688	0.1896	0.3240
FS	0.7628	0.1815	**0.0304***
DX	0.6903	0.3866	**0.0066***
YZ	0.2729	0.3183	**0.0108***
SY	0.1172	0.1215	0.4768
CP	0.4751	0.194	0.6275
MTG	0.4887	0.2756	0.3661
YQ	**0.0230***	0.1305	0.4254
PG	0.1175	0.3340	0.1277
MY	**0.0180***	0.1766	0.8392
HR	**0.0165***	0.0699	0.5287
DL	0.5362	0.1777	0.6535
YF	0.5453	0.7069	**0.0068***
MYSK	**0.0083***	0.0391	0.3552
BDL	**0.0072***	0.3128	0.6200
YLD	0.3262	0.2919	0.2011
LLH	0.5881	0.1673	0.1284
DGC	0.1654	0.8385	0.6367
YDMN	0.4368	0.1505	0.7003
NSH	0.396	0.6954	0.0656
DSH	0.3999	0.3104	0.0885
XZMB	0.1878	0.2025	0.1287
Mean	0.2275	0.1811	0.1093

* denotes that weekend and weekday difference is significant at the 90% confidence level by Wilcoxon rank-sum test

Number in bold denotes that weekend and weekday difference is significant at the 90% confidence level by student's *t*-test and Wilcoxon rank-sum test

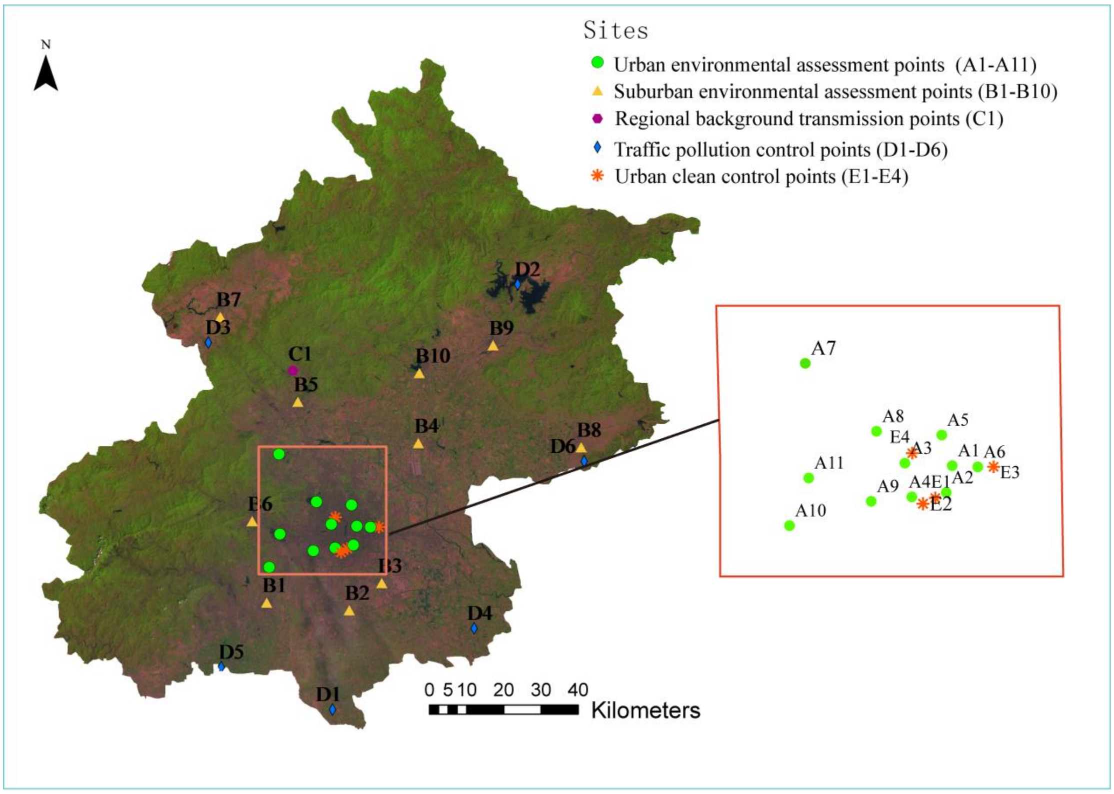

Supplementary Figure 1 Spatial distributions of 32 observation stations in Beijing

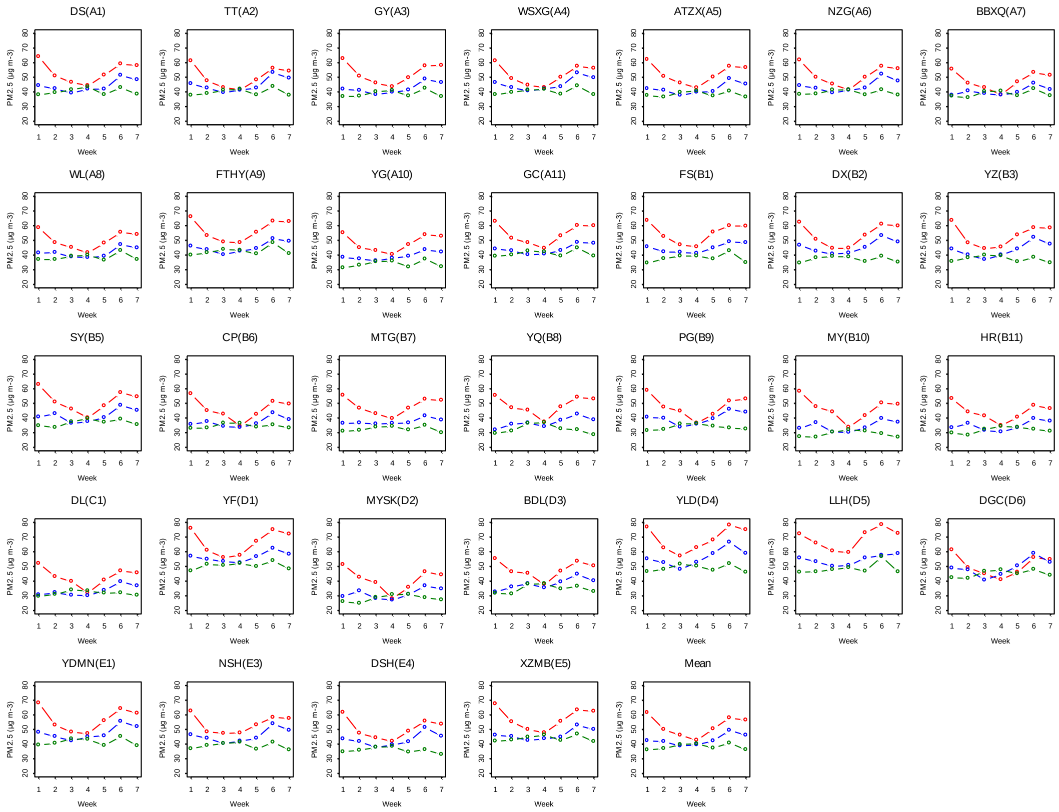

Supplementary Figure 2 $PM_{2.5}$ concentrations by day of the week. The annual average $PM_{2.5}$ concentrations by day of the week at 32 stations and the mean of 32 stations in Beijing in 2018 (red lines), 2019 (blue lines), and 2020 (green lines).

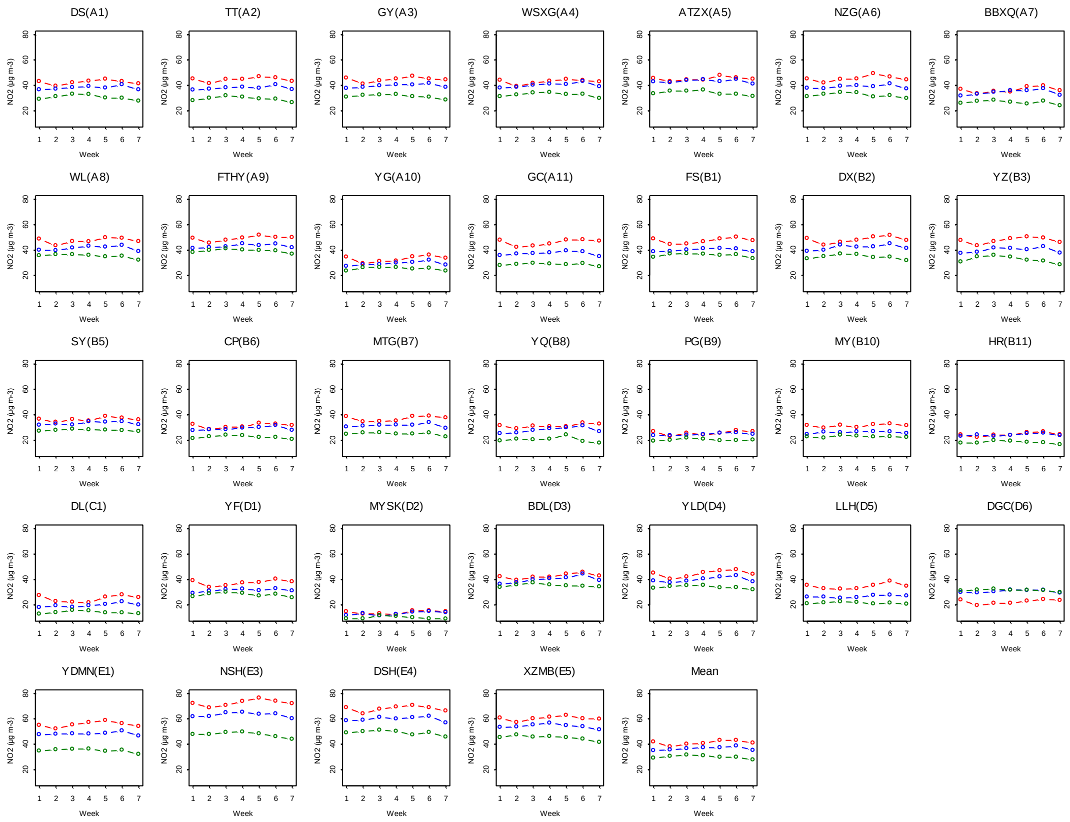

Supplementary Figure 3 NO_2 concentrations by day of the week. The annual average NO_2 concentrations by day of the week at 32 stations and the mean over 32 stations in Beijing in 2018 (red lines), 2019 (blue lines), and 2020 (green lines).

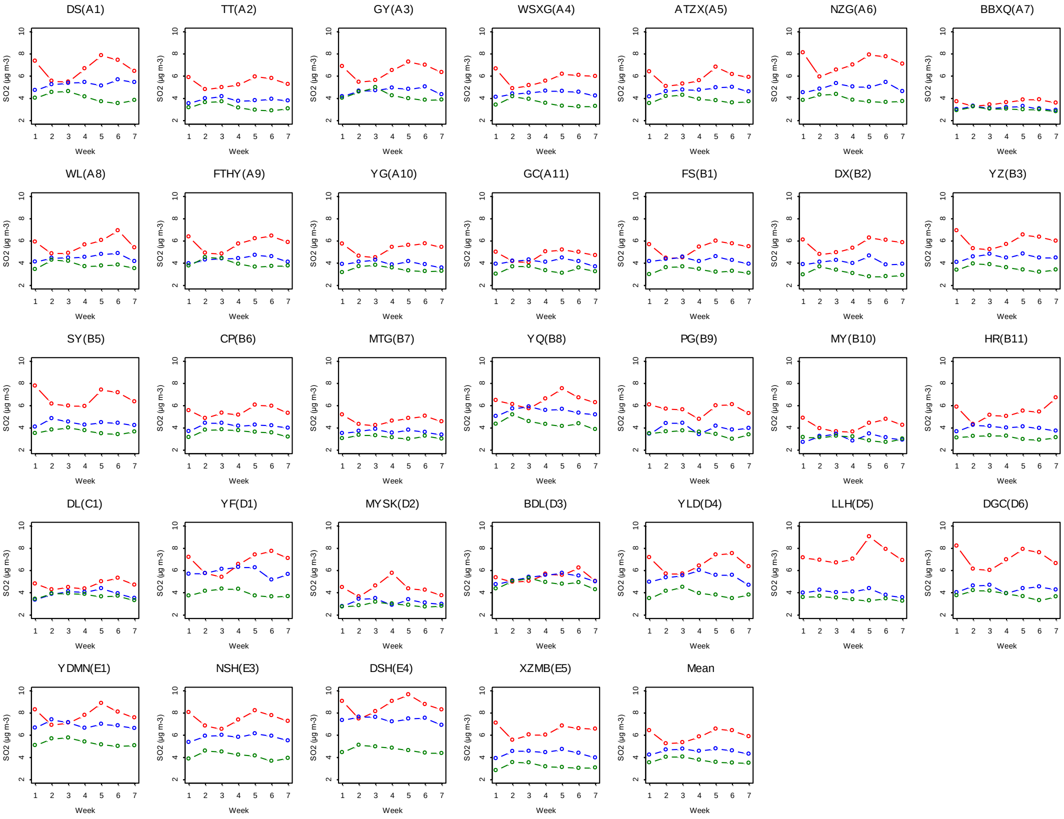

Supplementary Figure 4 SO_2 concentrations by day of the week. The annual average SO_2 concentrations by day of the week at 32 stations and the mean over 32 stations in Beijing in 2018 (red lines), 2019 (blue lines), and 2020 (green lines).

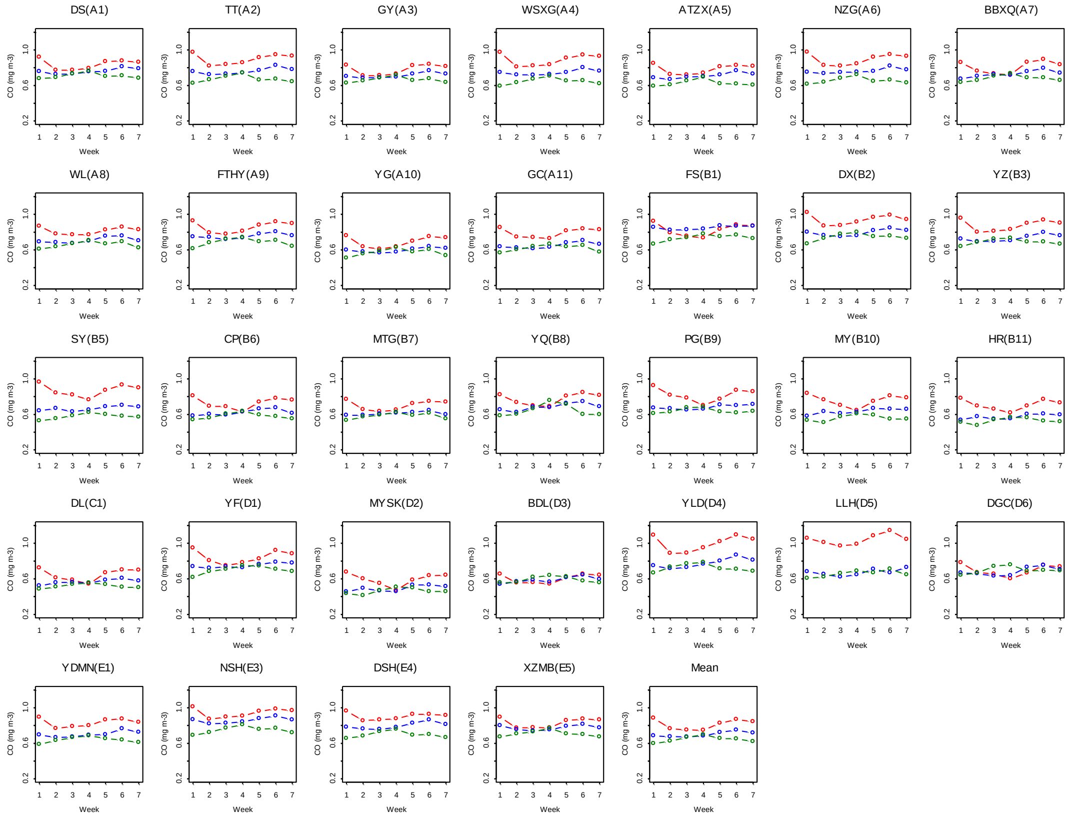

Supplementary Figure 5 CO concentrations by day of the week. The annual average CO concentrations by day of the week at 32 stations and the mean over 32 stations in Beijing in 2018 (red lines), 2019 (blue lines), and 2020 (green lines).

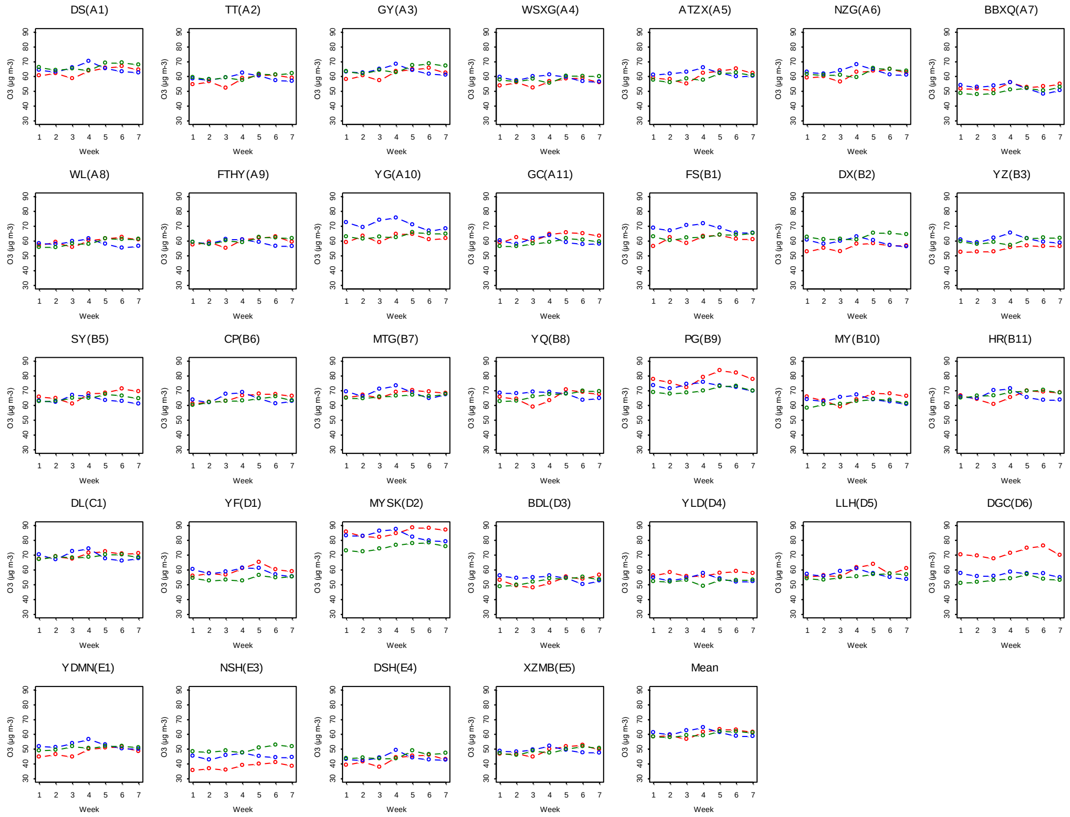

Supplementary Figure 6 O_3 concentrations by day of the week. The annual average O_3 concentrations by day of the week at 32 stations and the mean over 32 stations in Beijing in 2018 (red lines), 2019 (blue lines), and 2020 (green lines).

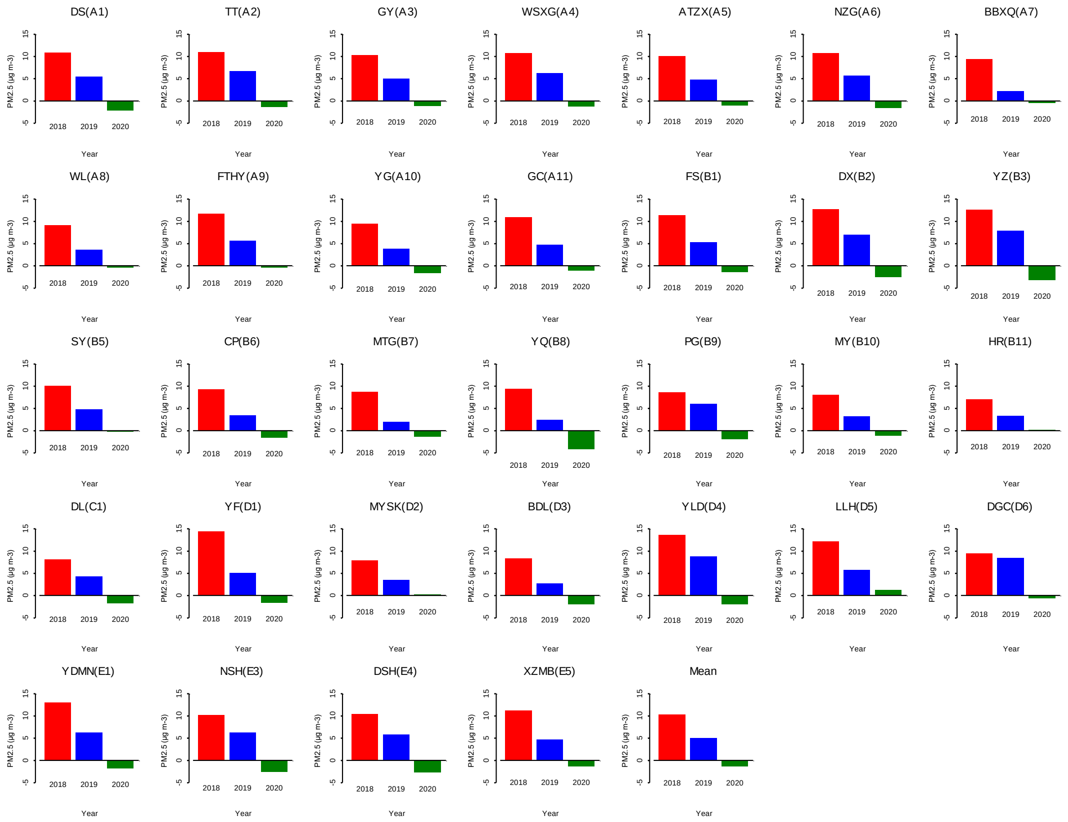

Supplementary Figure 7 The $PM_{2.5}$ weekend effect magnitudes. The $PM_{2.5}$weekend effect magnitudes at 32 stations and the mean over 32 stations in Beijing in 2018 (red bars), 2019 (blue bars), and 2020 (green bars). The weekend refers to Saturday through Sunday, and weekday is Tuesday through Friday. The weekend effect magnitude is computed by subtracting the average weekday value from the average weekend value.

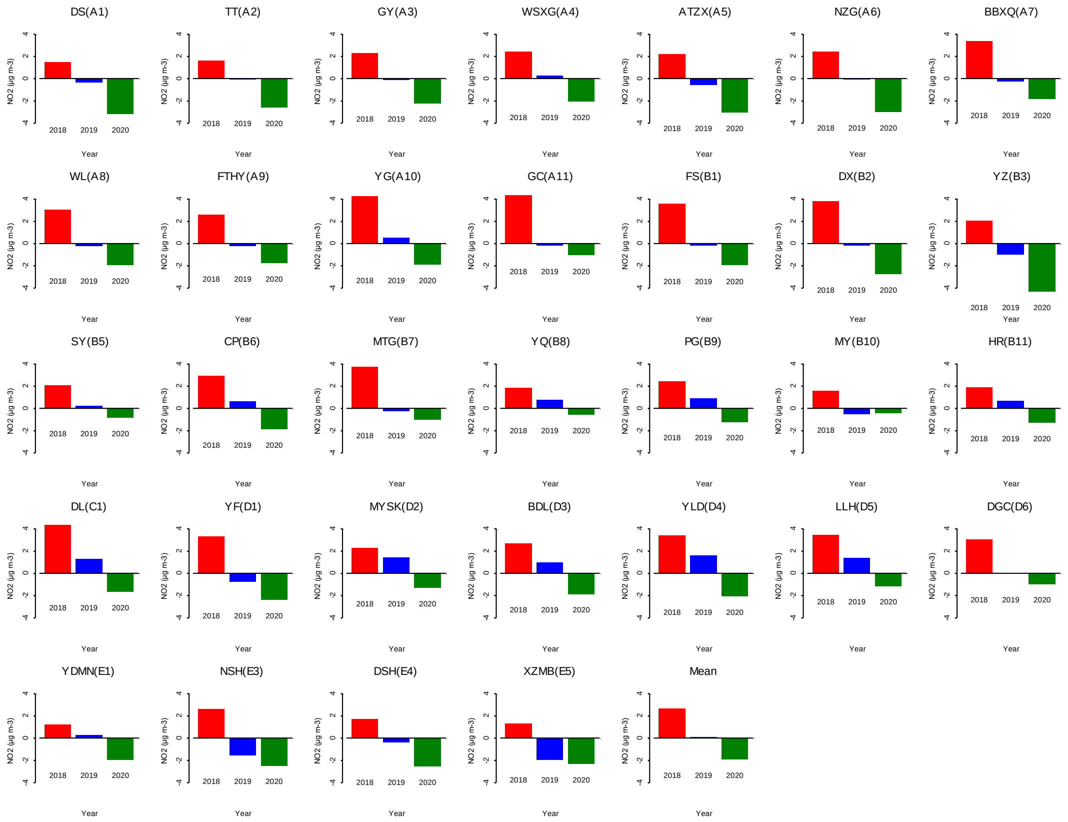

Supplementary Figure 8 The NO_2weekend effect magnitudes. The NO_2 weekend effect magnitudes at 32 stations and the mean over 32 stations in Beijing in 2018 (red bars), 2019 (blue bars), and 2020 (green bars). The weekend refers to Saturday through Sunday, and weekday is Tuesday through Friday. The weekend effect magnitude is computed by subtracting the average weekday value from the average weekend value.

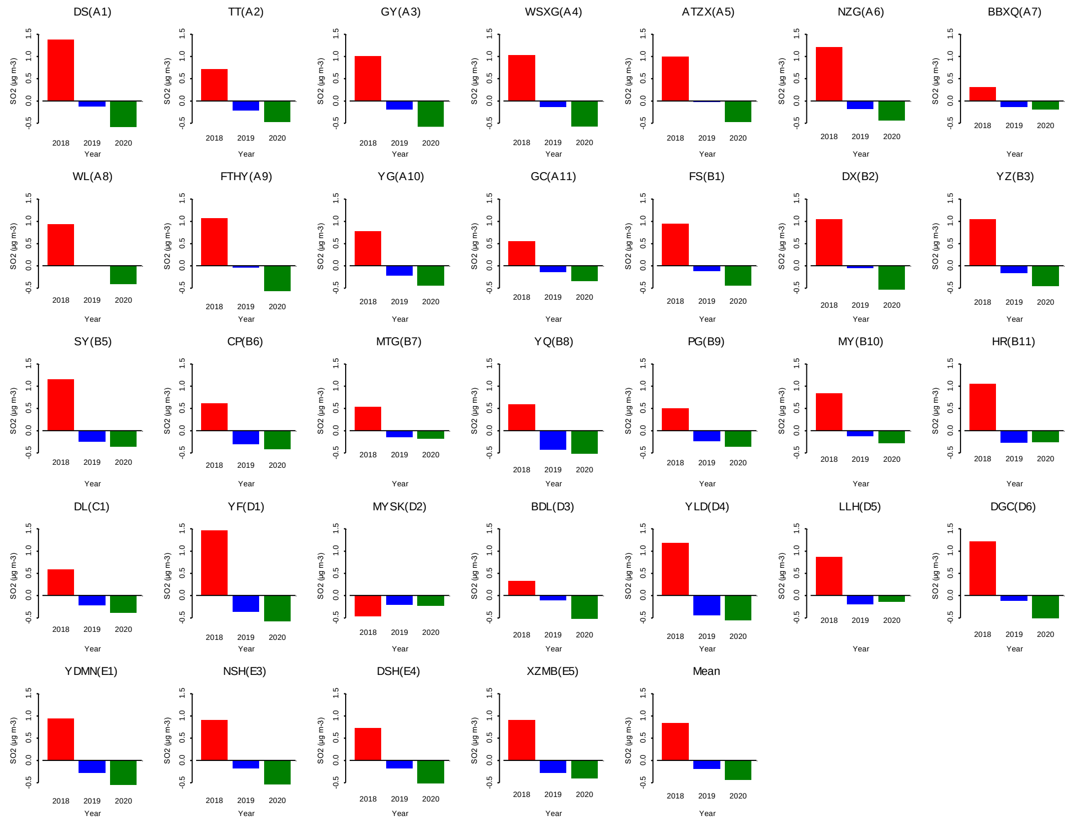

Supplementary Figure 9 The SO_2 weekend effect magnitudes. The SO_2 weekend effect magnitudes at 32 stations and the mean over 32 stations in Beijing in 2018 (red bars), 2019 (blue bars), and 2020 (green bars). The weekend refers to Saturday through Sunday, and weekday is Tuesday through Friday. The weekend effect magnitude is computed by subtracting the average weekday value from the average weekend value.

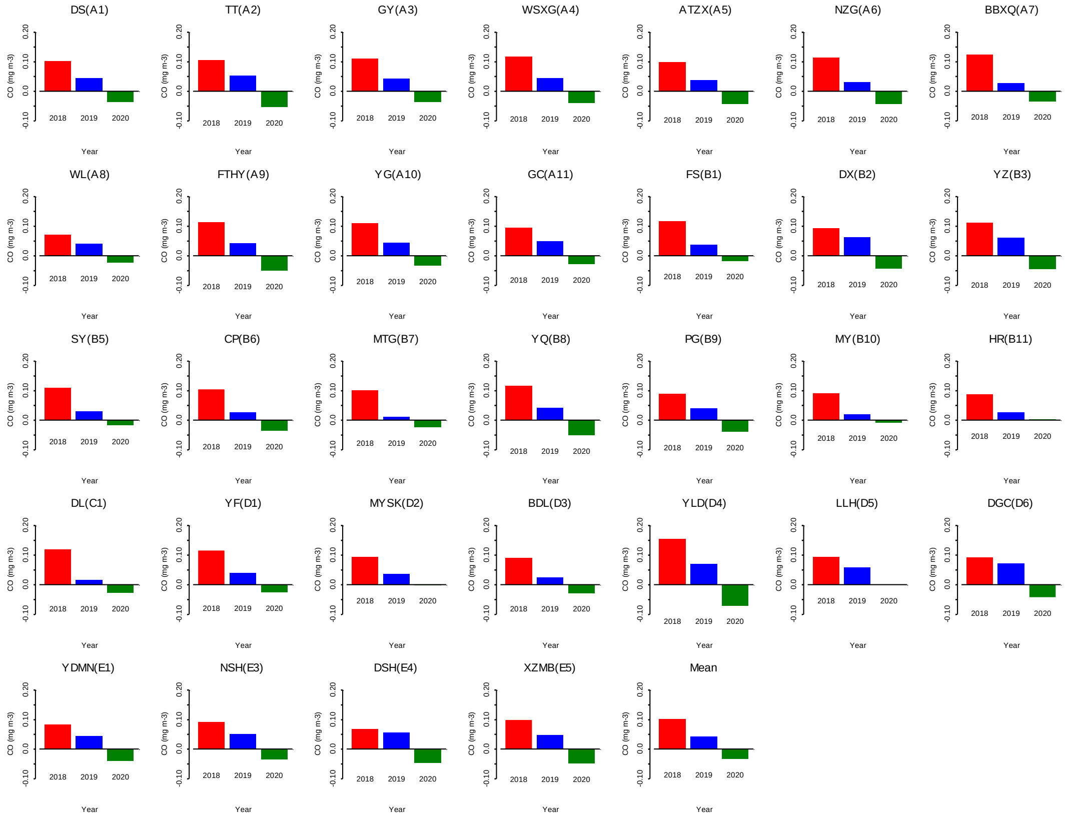

Supplementary Figure 10 The CO weekend effect magnitudes. The CO weekend effect magnitudes at 32 stations and the mean over 32 stations in Beijing in 2018 (red bars), 2019 (blue bars), and 2020 (green bars). The weekend refers to Saturday through Sunday, and weekday is Tuesday through Friday. The weekend effect magnitude is computed by subtracting the average weekday value from the average weekend value.

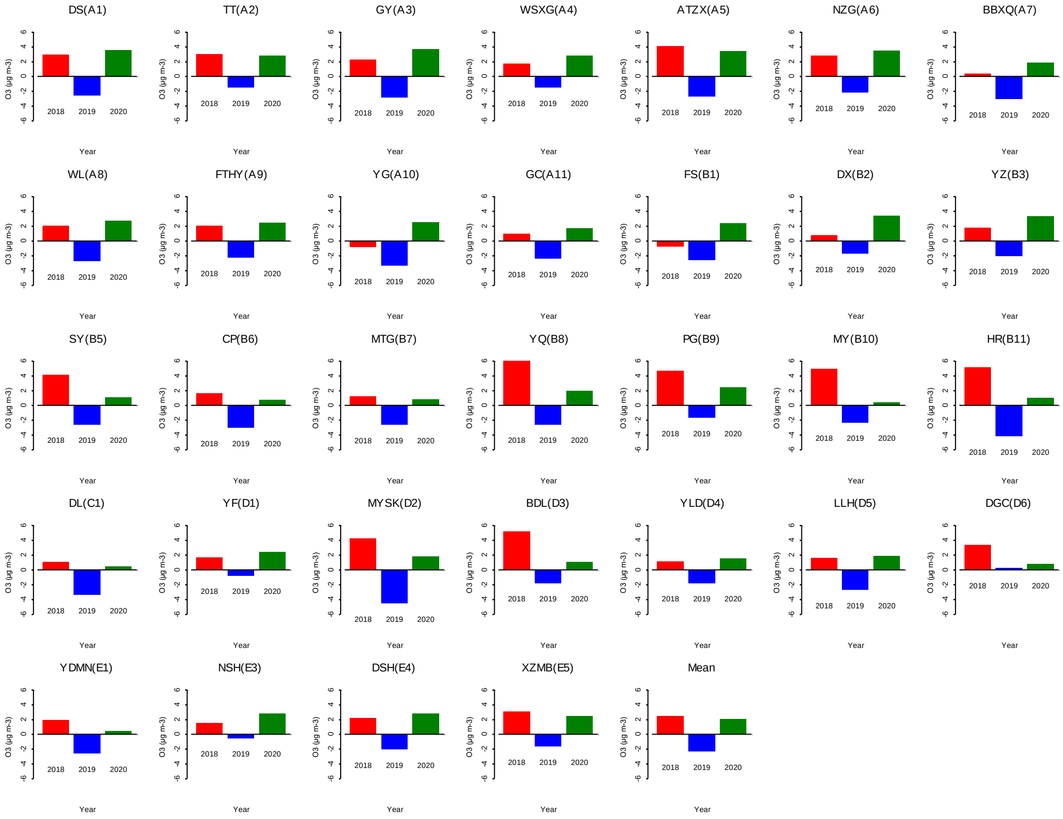

Supplementary Figure 11 The O_3 weekend effect magnitudes. The O_3 weekend effect magnitudes at 32 stations and the mean over 32 stations in Beijing in 2018 (red bars), 2019 (blue bars), and 2020 (green bars). The weekend refers to Saturday through Sunday, and weekday is Tuesday through Friday. The weekend effect magnitude is computed by subtracting the average weekday value from the average weekend value.

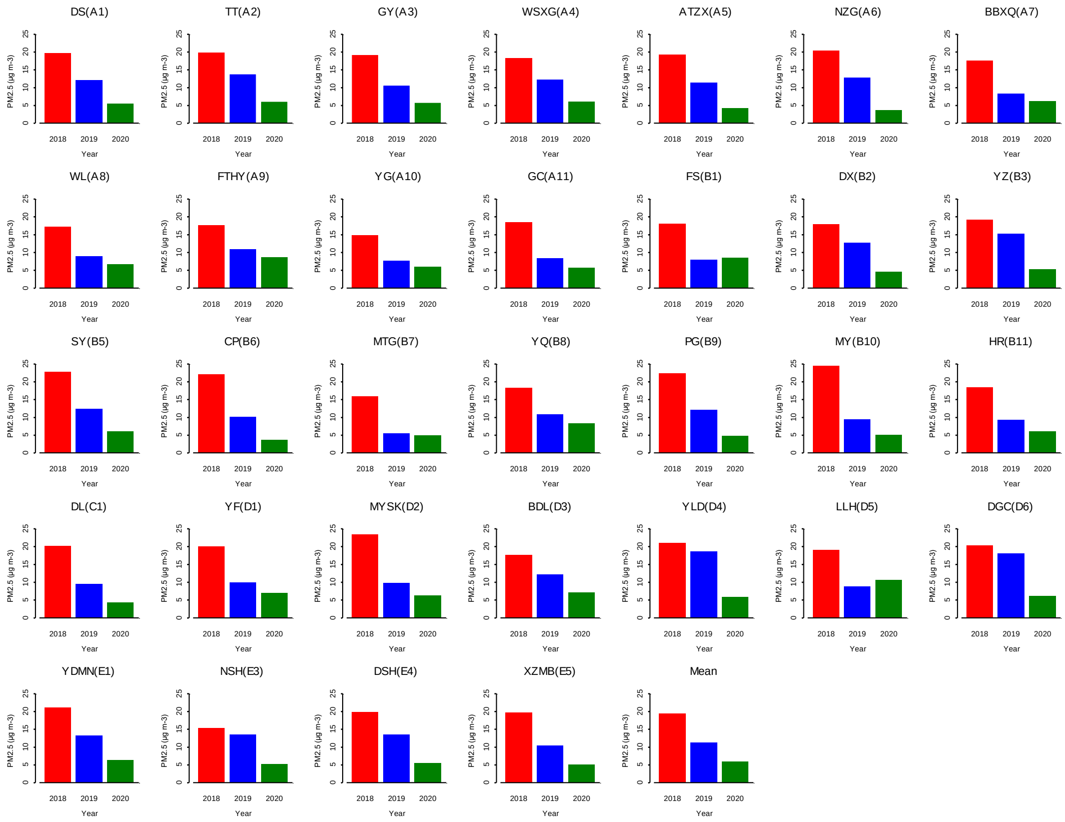

Supplementary Figure 12 The weekly cycle magnitudes of $PM_{2.5}$. The weekly cycle magnitudes of $PM_{2.5}$ at 32 stations and the mean over 32 stations in Beijing in 2018 (red bars), 2019 (blue bars), and 2020 (green bars). The weekly cycle magnitude is defined as the difference between the weekly maximum and minimum.

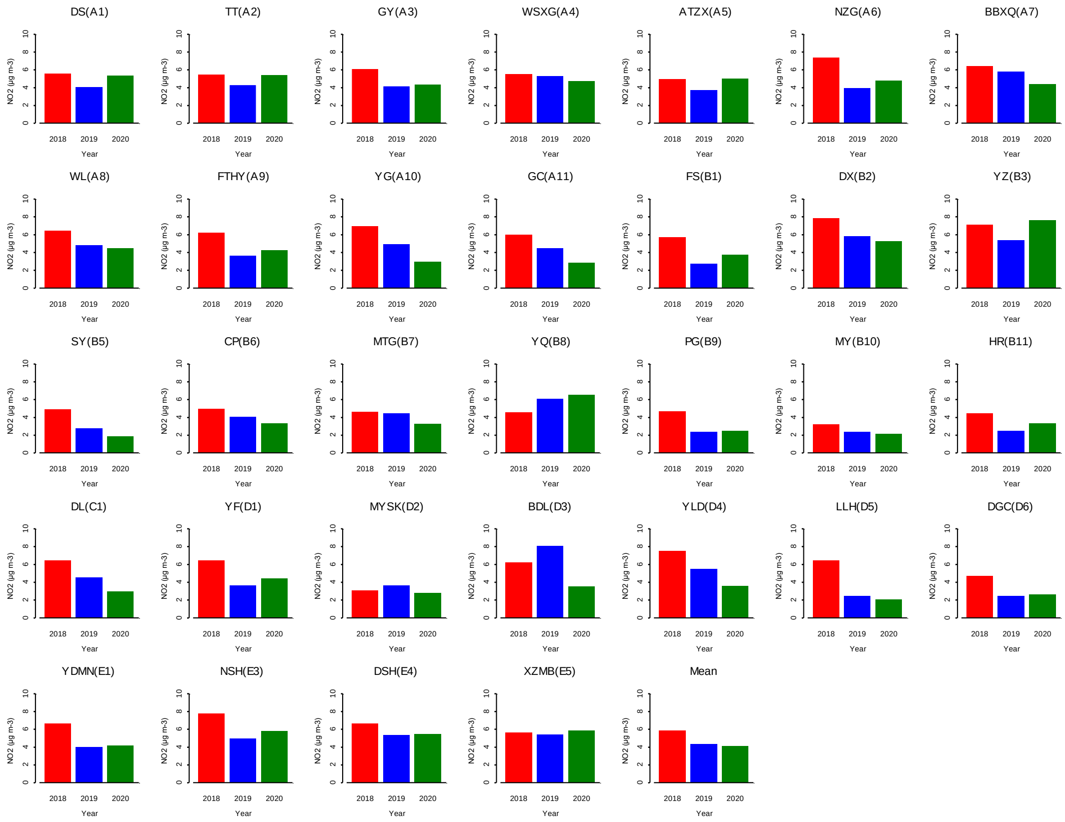

Supplementary Figure 13 The weekly cycle magnitudes of NO_2. The weekly cycle magnitudes of NO_2 at 32 stations and the mean over 32 stations in Beijing in 2018 (red bars), 2019 (blue bars), and 2020 (green bars). The weekly cycle magnitude is defined as the difference between the weekly maximum and minimum.

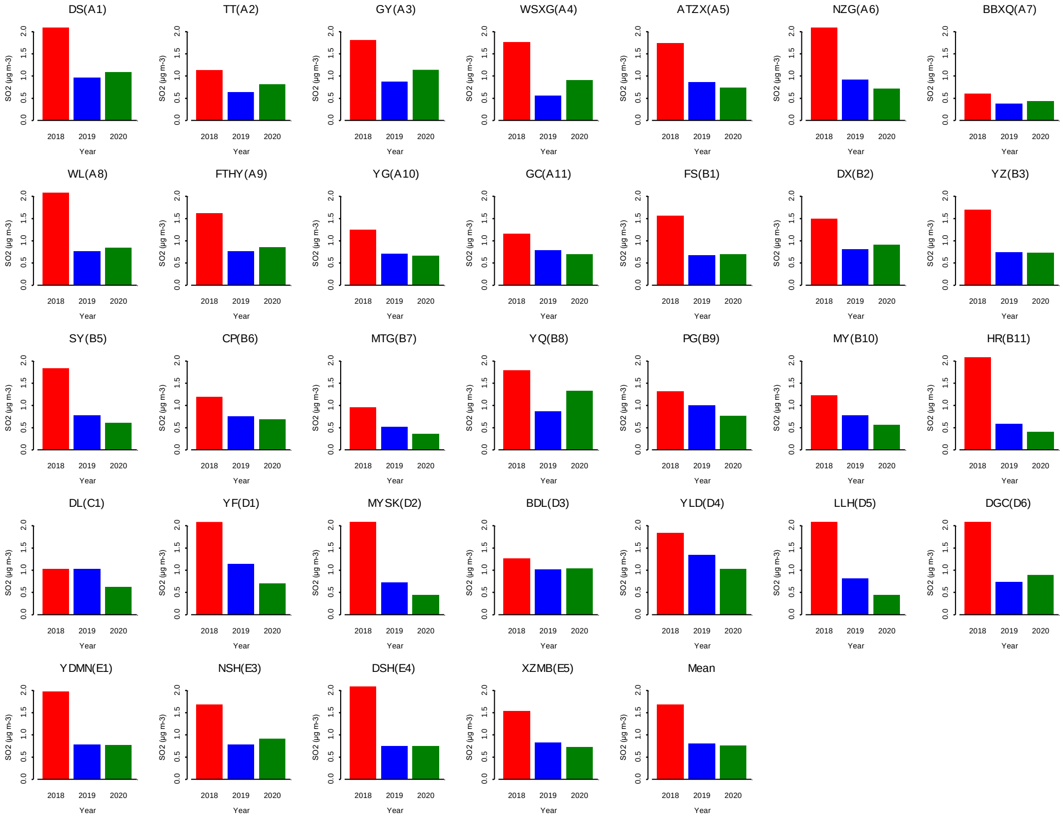

Supplementary Figure 14 The weekly cycle magnitudes of SO_2. The weekly cycle magnitudes of SO_2 at 32 stations and the mean over 32 stations in Beijing in 2018 (red bars), 2019 (blue bars), and 2020 (green bars). The weekly cycle magnitude is defined as the difference between the weekly maximum and minimum.

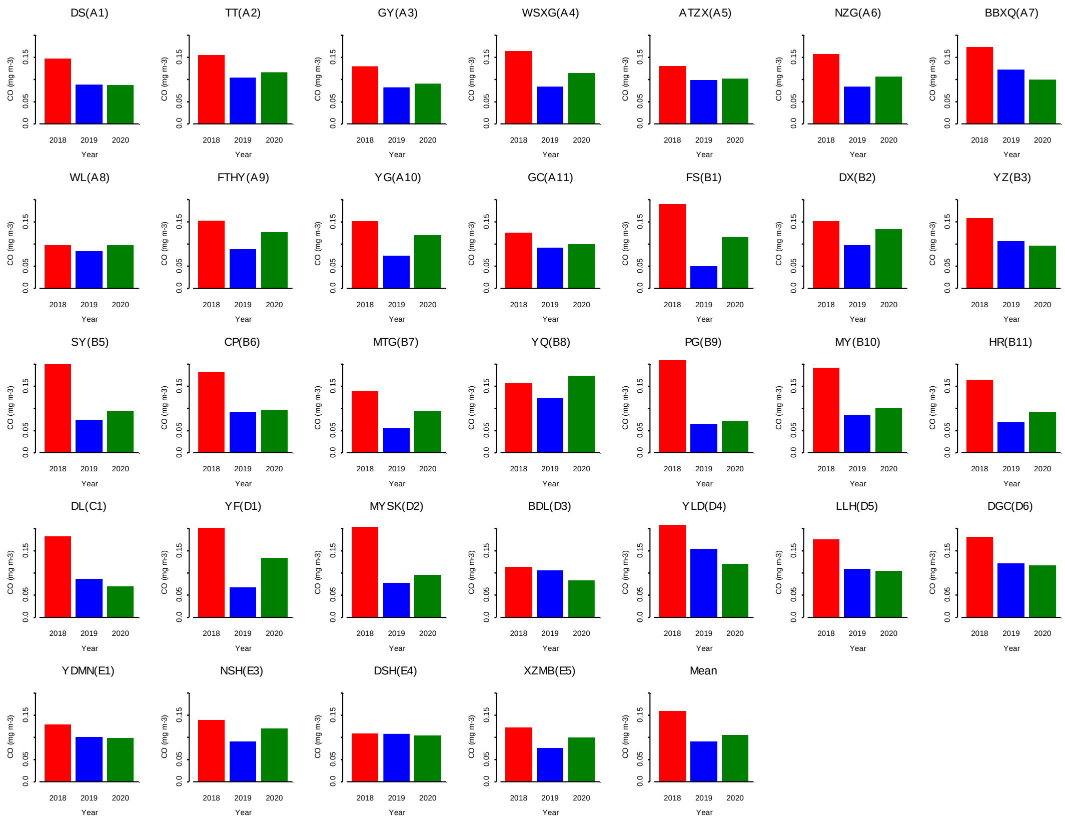

Supplementary Figure 15 The weekly cycle magnitudes of CO. The CO weekly cycle magnitudes at 32 stations and the mean over 32 stations in Beijing in 2018 (red bars), 2019 (blue bars), and 2020 (green bars). The weekly cycle magnitude is defined as the difference between the weekly maximum and minimum.

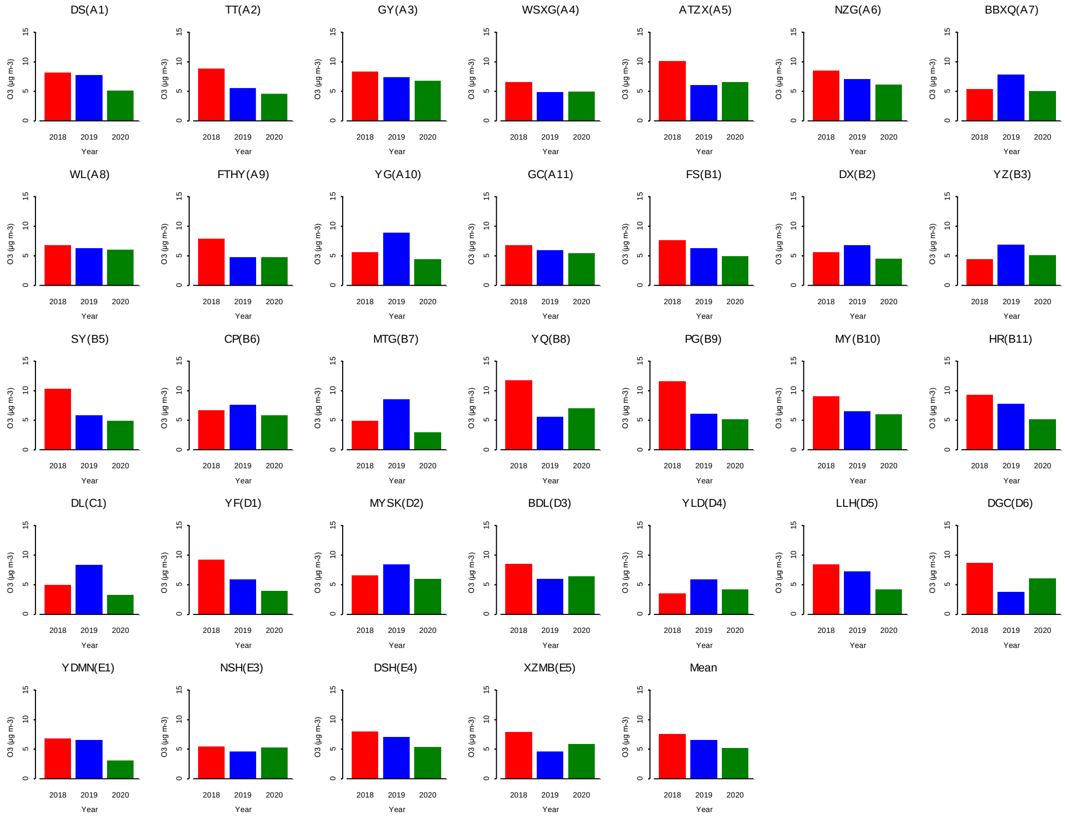

Supplementary Figure 16 The weekly cycle magnitudes of O_3. The weekly cycle magnitudes of O_3 at 32 stations and the mean over 32 stations in Beijing in 2018 (red bars), 2019 (blue bars), and 2020 (green bars). The weekly cycle magnitude is defined as the difference between the weekly maximum and minimum.
